# Rapid and spontaneous recovery in autistic disorder

**DOI:** 10.4103/0019-5545.55092

**Published:** 2009

**Authors:** Prabhat Sitholey, Vivek Agarwal, Amol Pargaonkar

**Affiliations:** Department of Psychiatry, CSM Medical University (Former K. G. Medical University), Lucknow - 226 003, India; 1 Department of Royal Perth Hospital, Perth, Australia

**Keywords:** Autistic disorder, child, spontaneous recovery

## Abstract

Recovery in autistic disorder is rare. There are few reports of recovery from autistic disorder after a few years of therapeutic intervention. We report here a case of autistic disorder who recovered spontaneously without any intervention in 13 days.

## INTRODUCTION

Pervasive developmental disorders (PDD) are chronic, lifespan disorders in which complete recovery, generally, does not occur. Symptoms of PDD, once they arise, do not remit. Some PDD symptoms may remit with age but others appear in their place so that the disorder persists. However, improvement can occur in PDD. More improvement is likely in Asperger's disorder as compared to autistic disorder.

Recovery in PDD may be variously defined. One way is to assess an individual with PDD, after some time of treatment, in terms of special services or provisions he requires, his ability to live independently, and his social adjustments as compared to his peers. If there is no significant difference, he may be considered as recovered. Another way is to see whether specific symptoms required for a valid PDD diagnosis are present or not. If some PDD symptoms are present but not enough for a valid diagnosis, the affected person may be said to have sub-syndromal PDD. If the diagnostic criteria for PDD were met earlier but not later, the patient may be called greatly recovered. If no specific PDD symptoms are present, recovery may be called complete. Complete recovery in PDD must be extremely rare. Our www.pubmed.gov and www.google.com search for authentic, scientific reports, in which specific autistic symptoms and their outcome is described, yielded only one case of Temple Grandin, who had Asperger's disorder and in autistic disorder, we could get only two reports, each of two cases, in which recovery took place.[1,2] In the latter four cases, recovery took place after 2-4 yrs of management.

Lovaas[3] and McEachin Smith and Lovaas[4] reported recovery in eight autistic children treated by Lovaas methods. Outcome of specific autistic symptoms was not described in these reports. Autism Research Institute (ARI) website, www.autism.com gives video reports of recoveries in some autistic children. These reports have not been reviewed. Therefore, it is difficult to be sure of the level of recovery in the above instances.

We report here a case of autistic disorder in which rapid and spontaneous recovery occurred without any specific therapeutic intervention in 13 days.

## CASE REPORT

P, a 5.6 yr old male child was brought by his parents with complaints of delayed development and being aloof most of the time for 3 yr. He was hospitalized on June 14, 2005 for management.

P's antenatal period was normal. He had delayed developmental milestones. As far as the parents could remember P was able to sit without support at 1 yr of age, walk without support at 3 yr. Social smile was seen at 5 months. P was able to indicate for toilet needs by 3 yr. He also started recognizing his parents by 3 yr. He started producing sounds by 3.5 yr and speaking monosyllables by 4 yr of age. P was vaccinated. The parents became worried about P's lack of emotional attachment with them and his two brothers when he was about 2.6 yr. He would not make meaningful eye-to-eye contact even when the family members tried to do so. Attempts to pick him up in lap, kissing his cheek with affection etc., did not elicit appropriate emotional or behavioral response. He always remained aloof. He never participated in other children's play or included them in his own. He could speak polysyllables but no meaningful word. He made no attempts to communicate by means of gestures. He would mouth, twist, or bang the toys against the floor. P preferred playing with pieces of paper, plastic bags, and polythene sachets and producing crackling sound by crumpling them. He tore paper to small bits and if prevented, he would become annoyed, shout, and cry. P rarely cried or showed his discomfort on being injured. His sleep pattern was irregular. He was hyperactive and would not sit at one place for long. He would go from room to room without any apparent reason.

P belonged to a nuclear, low-middle class urban family. P's physical examination including neurological assessment was within normal limits. On Vineland Social Maturity Scale (VSMS), his social/developmental age was 1.5 yr and SQ/DQ 25-30. On Childhood Autism Rating Scale (CARS) he had a score of 40.5 (severe autism) on June 16, 2005. P's hemogram and other laboratory investigations were normal. His CT scan of brain was normal. Cytogenetic assessment revealed normal male karyotype.

P was diagnosed as a case of autistic disorder with severe mental retardation (MR) as per DSM-IV-TR criteria.

P's behavior was being continuously observed in multiple settings. An improvement in his autistic symptoms was observed from June 23, 2005 onward. Observations on June 29, 2005 revealed age appropriate reciprocal social inteaction, communication by means of gestures, and adequate interest in toys. No evidence of any autistic symptoms was seen. P showed many remarkable behavior changes from before. He would now approach the persons known to him on his own and broadly smile at them. When called by name he would look at the caller and smile at him. He would make himself comfortable in his mother's lap, hug her affectionately, and mould his body according to her. He would catch hold of his father's trousers and hug his legs. He would now not go away from his parents, doctors, nurses, and occupational therapist. P's parents and we (PS, VA and AP) could feel his emotional warmth. P's parents especially noted this remarkable emotional change and were delighted at this. P started playing with a rubber ball and toys like a plastic truck, an elephant with wheels, and dolls. He would show these toys to others and seek their help in playing with them. When AP threw the ball toward P, he would roll it back to AP. He would no longer bang or mouth the toys or use them inappropriately. He was now not interested in pieces of paper or polythene sachets. P now followed verbal commands. He could be taught to shake hands with AP and play peek-a-boo. He could point out objects. The parents noted that P expressed his discomfort and pain more clearly than before. From his facial expression, body language, and crying they could easily make out his distress as compared to his previous relative indifference to pain. His eye-to-eye contact remained transient but it was without indifference for others.

P had a score of 18 (non-autistic) on CARS on June 29, 2005. In view of the above observations and the fact that P had been given a confident diagnosis of autistic disorder 13 days back it was thought that he had achieved a rapid, spontaneous, and complete recovery from autistic disorder.

P remained hospitalized for further 6 weeks. His repeat SQ/DQ on VSMS on August 2, 2005 was 40-45. P was rehospitalized from December 6-10, 2005 for psychiatric assessment. All the psychosocial gains earlier made by P persisted and he was found to be more affectionate, interactive, and playful. His performance IQ on Seguin form Board Test was 55-60 and mental age was 3.5 yrs. P was periodically followed up to March 3, 2008. P did not show any autistic symptoms. On examination on March 3, 2008, P's emotional warmth, his bright appearance, cheerful smile, his social and emotional relatedness, normal meaningful but transient eye contact and moderate- to- severe hyperactivity and short attention span, and constantly trying to say something were most striking. P, now 9.2 yr, mostly spoke single meaningful words and at times combined two words together in a small phrase. He could unbutton take off, put on and button his shirt. He could only scribble and draw a curvy vertical line when asked to imitate a drawn vertical line. According to the parents he can eat and drink independently. He can partially clean himself after defecation and requires help. He could carry out simple commands like ‘go and fetch objects’. P could uncap a water bottle, drink from it and cap it. He spontaneously came forward to meet and shake hands. He spontaneously offered peanuts to a resident. He could point to the objects. P spoke in the mobile phone in a make believe address to an imaginary person.

## DISCUSSION

P completely recovered from autistic disorder while hospitalized and under our continuous observation. It may be argued that the diagnosis of autistic disorder was wrong. However, in P typical autistic symptoms were continuously present for at least 3 yr with onset before age 3. These were directly observed by the multiple observers in the multiple settings. PS, VA, and AP made a confident diagnosis by consensus. Besides, P had a score of 40.5 on CARS denoting severe autism. Therefore, there is no doubt about the diagnosis of autistic disorder in P.

P recovered completely without any specific intervention for autistic disorder. In autistic disorder, the presence of some communicative speech by age 5 and performance IQ more than 50 is related to better prognosis.[5] P had SQ/ DQ 25-30 and no communicative speech, which indicate worse prognosis, and yet P made a rapid and spontaneous recovery without any specific treatment.

From a psychosocial point of view, historically there was no deprivation, lack of love, communication, stimulation or neglect in the family. On our observation, we found both the parents to be normally concerned, very patient, loving and caring. Besides, historically, P's other two sibs were normal. Therefore, P's autistic disorder was due to neither adverse psychosocial circumstances nor his recovery from it due to any changes in them.

It might be thought that hospitalization was responsible for recovery of P from autism. Such a recovery would be expected in a child who was neglected or abused and deprived of love, affection, physical and psychological care, and of psychosocial stimulation. As mentioned above, P came from a loving and caring family very much concerned about P's development. His parents gave him adequate time required by his lag in development despite his autistic aloofness. While P was in the hospital, his parents remained his primary caretakers and everything in the hospital was done through them except for play and observation sessions in which the parents were mostly present. Thus, hospitalization was only a geographical and physical change for P unlikely to have effect upon P's autism. P did receive attention from the authors, occupational therapist, and the nurses during hospitalization but this was routine and not more than that for any other child inpatient. In past, our other autistic child inpatients had not recovered with such attention. In our opinion, it could not possibly have reversed P's autism in such a short time in any way. Thus, hospitalization does not explain recovery from autism.

We have not come across any other instance in which recovery took place spontaneously and so rapidly. In the two cases described by Gajzago and Prior,[1] therapeutic intervention in terms of medication (although without benefit), behavior modification, and education was provided by the family and teachers. Both did not have MR. Both acquired some communicative speech before age 5. In both cases, recovery took 3-4 yr. Similarly, the two siblings described by Perry, Cohen, and Decarlo[2] were diagnosed as autistic by age 2 and after about 1.5-2 yr of behavior therapy and education; they improved and were non-autistic by age 4. The siblings initially had developmental delays, which improved with the improvement in autistic symptoms.

Thus, in our view, P's recovery from autistic disorder is unexpected and unexplained. The above five cases (four of the above two case reports and one of P) show that recovery is possible even in cases of autistic disorder. However, in these children recovery occurred during early childhood, before age 6. It is not known whether such recoveries could also occur later during school age, adolescence, or adulthood. The recoveries may take a few years as in above four cases or about a fortnight as in P Although, such recoveries must be extremely rare, these need to be reported. The families of autistic children, the clinicians, and therapists dealing with childhood autism must know that this can happen.
